# Cellulose-Rich Polysaccharide Extracts with Gel-Forming Potential and Improved Antioxidant Properties from Stem (*Vitis vinifera* L.) By-Products: Ultrasound-Assisted Aqueous Extraction and Characterization

**DOI:** 10.3390/gels12020154

**Published:** 2026-02-09

**Authors:** Francesca Comas-Serra, Valeria S. Eim, Rafael Minjares-Fuentes, Víctor M. Rodríguez-González, Antoni Femenia

**Affiliations:** 1 Department of Chemistry, University of the Balearic Islands, Ctra Valldemossa Km 7.5, 07122 Palma de Mallorca, Spain; xesca.comas@uib.es (F.C.-S.); valeria.eim@uib.es (V.S.E.); 2 Facultad de Ciencias Químicas, Universidad Juárez del Estado de Durango, Av. Artículo 123 s/n Fracc. Filadelfia, Gómez Palacio 35010, Durango, Mexico; rafael.minjares@ujed.mx (R.M.-F.); vicrogfcq7@ujed.mx (V.M.R.-G.)

**Keywords:** grape stems, ultrasound-assisted extraction, cellulose-rich polysaccharides, gel-forming extracts, antioxidant capacity, phenolics, green extraction, waste valorization

## Abstract

The valorization of wine by-products aligns with circular bioeconomy principles. This study investigates the ultrasound-assisted aqueous extraction (UAE) of bioactive compounds and cell wall polysaccharides from Syrah grape stems (*Vitis vinifera* L.) to produce polysaccharide extracts with the intrinsic potential to form cellulose-rich gels with enhanced antioxidant properties. Extractions were performed at three temperatures (10, 20, and 50 °C) and three ultrasonic power densities (120, 206, and 337 W/L), and compared to conventional extraction (CE, 200 rpm). The results demonstrated that UAE significantly accelerated the extraction kinetics for total phenolics (TP), flavonols, and antioxidant capacity (ABTS, FRAP), achieving up to a 3.1-fold increase in TP yield at 20 °C. Notably, UAE at 337 W/L and 20 °C produced antioxidant levels equivalent to those obtained by CE at 50 °C, enabling high efficiency at lower, compound-preserving temperatures. Carbohydrate analysis revealed that the extracts were inherently “cellulose-rich” (glucose ~49–52 mol%), with co-extracted pectins and hemicelluloses constituting a composite polysaccharide matrix with inherent gel-forming capacity, as evidenced by its composition. While total polysaccharide yield was maximized at 10 °C, UAE’s primary effect was the facilitation of extraction and potential structural modification of polymers rather than increasing bulk yield. The process reduced extraction times by 3- to over 6-fold to achieve equivalent bioactive yields compared to CE. This work establishes UAE with water as a process aligned with green chemistry principles, an efficient strategy for the integrated, one-step recovery of antioxidant phenolics and gel-forming polysaccharides from grape stems, transforming this underutilized residue into a multifunctional extract precursor for cellulose-rich hydrogels suitable for food and pharmaceutical applications.

## 1. Introduction

The transformation of agri-food waste into high-value products is a cornerstone of the circular bioeconomy, directly supporting global sustainability goals aimed at waste reduction and resource efficiency [1,2]. The wine industry, a significant contributor to agricultural residues, generates approximately 5–9 million tons of by-products annually worldwide, with grape stems constituting up to 30% of this stream [3,4]. Traditionally regarded as low-value waste, grape stems (*Vitis vinifera* L.) are now recognized as a rich source of bioactive compounds, particularly dietary fiber, comprising the structural cell wall polymers cellulose, hemicelluloses, and pectins, and a diverse profile of phenolic antioxidants [5,6,7]. While the recovery of soluble phenolics has been extensively documented, the simultaneous extraction and functionalization of the insoluble cell wall matrix into advanced biomaterials, such as hydrogels, presents a more complex and underexplored opportunity for valorization [8,9].

Conventional thermal extraction methods often fail to efficiently liberate these tightly integrated polysaccharide networks without inducing degradation through mechanisms like pectin β-elimination, which compromises their gelling and structural integrity [10]. Furthermore, such processes frequently require organic solvents, raising environmental and safety concerns for food or pharmaceutical applications [11]. Water, as a green, non-toxic solvent, is ideal for extracting biocompatible polymers; however, its efficiency is intrinsically limited by the native insolubility and robust supramolecular associations (hydrogen bonding, hydrophobic interactions) within the lignocellulosic matrix [12]. This challenge necessitates innovative, non-thermal processing technologies. Ultrasound-assisted extraction (UAE) has emerged as a transformative solution, where the physical forces generated by acoustic cavitation, including microjet implosion, shear stress, and cell wall fragmentation, mechanically disrupt the plant tissue [13]. This action significantly enhances mass transfer and the release of high-molecular-weight functional polysaccharides into aqueous media, often while better preserving their native structure compared to prolonged thermal treatments [14,15].

Critically, contemporary research indicates that UAE’s role extends beyond merely improving extraction yield. It can induce targeted structural modifications in the extracted biopolymers [16]. For instance, controlled ultrasonic depolymerization of pectin can modulate its molecular weight and branching, influencing both its gelation kinetics and its ability to interact with other compounds [10,17]. This presents an opportunity to use UAE not only for extraction but as a green processing tool to in situ create and nanostructure composite biomaterials directly from biomass, where the modified biopolymer network serves as a multifunctional scaffold [18,19]. This scaffold could provide the physical matrix for a hydrogel while simultaneously enhancing the stabilization, bioavailability, and synergistic activity of the native phenolic antioxidants from the grape stems [20,21].

Despite the recognized potential of grape stems as a dual source of gelling polymers and antioxidants, a critical knowledge gap persists [22]. No prior study has systematically deconvoluted how specific aqueous UAE parameters (power density, temperature, time) directly govern the compositional profile of the extracted polysaccharide matrix—defining its “cellulose-rich” character—and how this profile, in turn, dictates the rheological and antioxidant performance of the resulting gel. The precise relationship between ultrasonic process conditions, the polysaccharide fingerprint (e.g., cellulose/pectin ratio, molecular weight distribution), and the emergent bioactive functionality of the final material remains unexplored.

Therefore, this study aims to bridge this gap by establishing a cause–effect framework for process optimization. The primary objective was to investigate and optimize the aqueous ultrasound-assisted extraction of grape stem by-products for the one-step production of precursor extracts designed for cellulose-rich gels with enhanced antioxidant capacity. The effects of ultrasonic power density, temperature, and time were systematically evaluated on two pivotal outcomes: (i) the yield and detailed monosaccharide composition of the extracted cell wall polysaccharides, providing a molecular basis for the gel’s structure; and (ii) the total phenolic content, flavonoid profile, and in vitro antioxidant activity (via ABTS and FRAP assays) of the integrated extracts/gels.

Thus, this work provides a scientific foundation for the sustainable valorization of grape stems, proposing a green extraction strategy consciously designed to tailor both the structural and bioactive properties of a novel, multifunctional hydrogel.

## 2. Results and Discussion

### 2.1. Carbohydrate Composition

The carbohydrate composition of polysaccharides extracted from *Vitis vinifera* L. stems under varying temperatures (10, 20, and 50 °C) and extraction methods (Conventional Extraction, CE; Ultrasonic-Assisted Extraction, UAE) is presented in Table 1. This compositional profile is critical for understanding the gel-forming potential of the extracts described in this work, as it directly reflects the polysaccharide networks solubilized from the lignocellulosic matrix and their response to processing.

The total sugar yield, corresponding to the mass of solubilized cell wall polymers, exhibited a significant inverse relationship with extraction temperature, decreasing from approximately 18.3–19.2 g/100 g fresh weight at 10 °C to 14.7–16.7 g/100 g at 50 °C [23]. This indicates that milder temperatures (10 °C) are optimal for maximizing extractable polysaccharide mass, a fundamental parameter for gel yield. While increased temperature typically enhances diffusion, this inverse trend aligns with the thermal degradation of gel-forming pectins via β-elimination, which can depolymerize chains and reduce their solubility [24,25]. Similar losses in soluble dietary fiber under thermal processing have been reported for other plant by-products [26]. Notably, UAE did not increase the total polysaccharide yield over CE at any temperature. UAE’s primary effect was to facilitate the extraction and solubilization of a specific polysaccharide network from the grape stem matrix. The altered extraction kinetics and final yield profiles are consistent with the potential for ultrasonic treatment to induce polymer modifications, which could be key for subsequent gel functionality [27].

The molar sugar composition provides the molecular rationale for the “cellulose-rich” character of the obtained gels, a notable finding for aqueous, non-alkaline extraction. The most consistent trend is the high and stable glucose (Glc) content (~49–52 mol%) across all conditions (Table 1). As noted by González-Centeno et al. [28], cellulose is the predominant cell wall polymer in grape stems (40–49% of total cell wall polysaccharides). Since crystalline cellulose is water-insoluble, this extracted glucose predominantly originates from non-crystalline, soluble β-glucans and hemicellulosic glucans (e.g., xyloglucans) that are intimately associated with cellulose microfibrils [29]. The efficient co-extraction of these polymers by both UAE and mild CE successfully liberates the cellulose-linked network, providing the foundational polymeric backbone for the observed gel structure. The minor fraction of 1M-Glucose further supports the presence of these hemicellulosic glucans. It is critical to clarify that gel formation from these extracts is a two-step process: (1) concentration of the extract is required to achieve a critical polymer concentration, and (2) gelation is typically induced by the addition of cross-linking ions, e.g., Ca^2+^ ions, leveraging the low methyl-esterification of the co-extracted pectins. The formation of a cohesive gel from these extracts therefore requires post-extraction concentration of the polymers, followed by ionic cross-linking [28,29].

The pectic components, indicated by uronic acids (Uronic A.), galactose (Gal), arabinose (Ara), and also rhamnose (Rha), showed clear sensitivity to processing. The data reveals a U-shaped trend for uronic acids versus temperature, with minima often observed at 20 °C, suggesting sub-optimal solubilization at this point and potential degradation at 50 °C. The slightly lower uronic acid yield with UAE at lower temperatures hints at ultrasonic cleavage of the homogalacturonan backbone, potentially modulating pectin molecular weight [30]. This is significant because, according to González-Centeno et al. [28], grape stem pectins are low methyl-esterified (Degree of Methyl-Esterification, DME: 21–39%), which influences their calcium-mediated gelation potential. This controlled depolymerization could enhance the gel’s bioactivity by increasing the availability of bioactive pectic oligosaccharides or improving the matrix’s capacity to retain phenolic compounds.

The stable xylose (Xyl) signal (~15 mol%), alongside mannose (Man) and fucose (Fuc), reflects the co-extraction of robust hemicelluloses like xylans and mannans. In grape stems, these polymers are deposited during secondary cell wall thickening alongside lignin [28]. Their presence is crucial, as they can act as cross-linking agents between cellulose microfibrils and the pectin network, directly contributing to the viscoelastic and hydration properties of the composite gel [31]. The variation in mannose content, particularly at 50 °C, suggests a differential extraction sensitivity for mannan-containing hemicelluloses depending on the method.

Our data, contextualized by prior characterizations [28], support a model where the extract possesses a ‘cellulose-rich’ polysaccharide profile, constituting a composite matrix with the intrinsic capacity to form a hydrogel. The high glucose content provides the structural backbone and thermal stability. The modulated pectin fraction, with its low DME, governs ionic gelation and hydration. The hemicellulose fraction facilitates cross-linking and matrix integrity.

The finding that UAE at low temperature (10 °C) provides the optimal compromise, maximizing total polymer yield while employing a physical method that can modify polymer interactions without thermal degradation, is pivotal. This strategy aligns with green extraction principles, preserving high-molecular-weight polymers necessary for gelation while potentially enhancing bioactivity through structural modification [27]. Consequently, the improved antioxidant properties likely arise not from a single compound but from the combined presence of rapidly liberated phenolics within a specific polysaccharide matrix. The modified extract matrix is structured to effectively interact with these antioxidants, resulting in the observed high activity.

### 2.2. Extraction Kinetics and Process Efficiency: Phenolics, Flavonols and Antioxidant Capacity

The influence of key operational parameters, such as extraction time, temperature, and ultrasonic power density, on the recovery of total phenolics (TP), flavonols, and antioxidant activity (ABTS, FRAP) was profound. This confirmed their critical role in the experimental design for comparing ultrasound-assisted extraction (UAE) with conventional extraction (CE). Aqueous extractions were therefore conducted across a temperature gradient (10, 20, and 50 °C) for up to 15 min using CE (200 rpm) and UAE at three acoustic power densities (120, 206, and 337 W/L).

**Figure 1:**
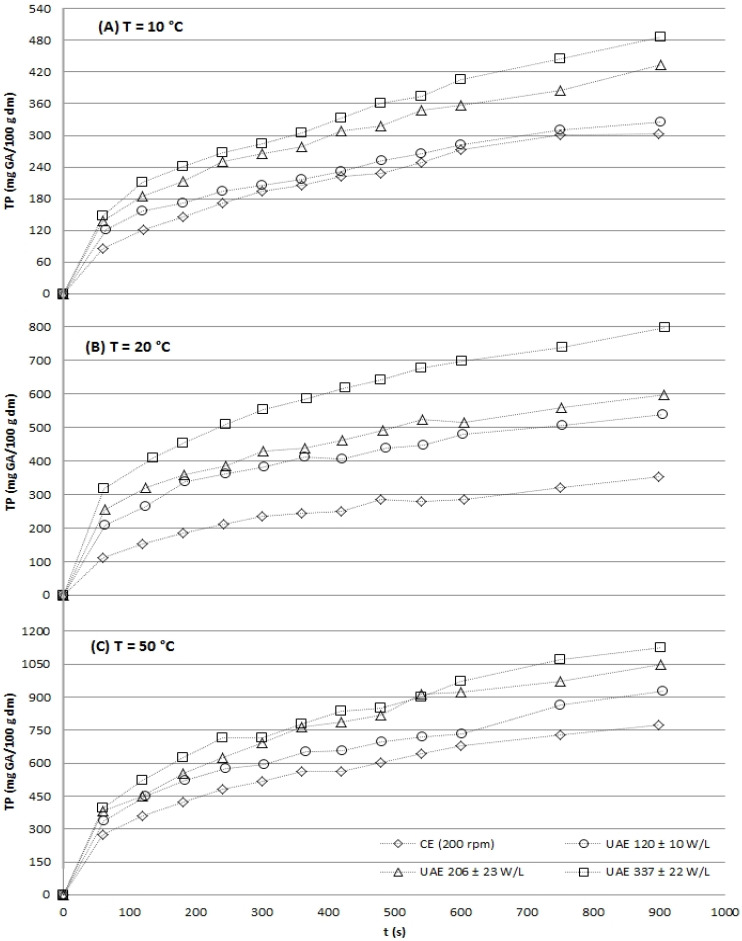
Aqueous experimental extraction kinetics of total phenolics (TP) from grape stems for both conventional (CE, 200 rpm) and UAE (at three acoustic power densities: 120 ± 10, 206 ± 23 and 337 ± 22 W/L) modalities for the three temperatures: 10 ± 1 °C (**A**), 20 ± 2 °C (**B**) and 50 ± 4 °C (**C**) in study.

**Figure 2:**
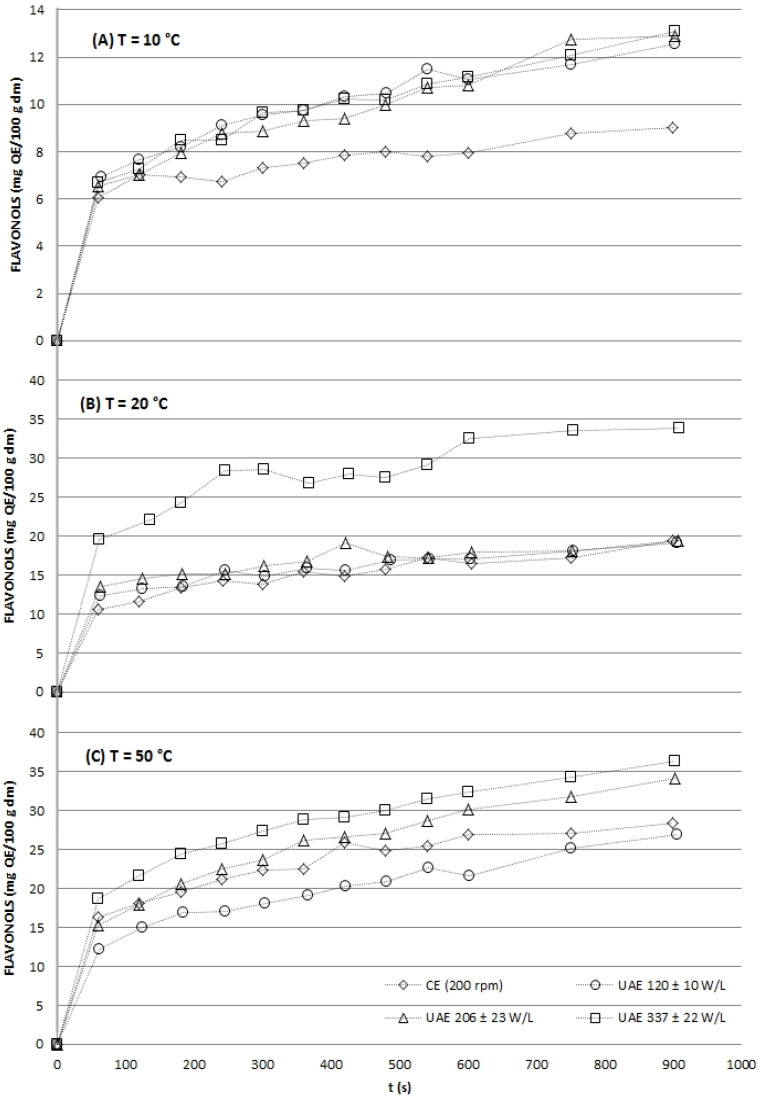
Comparison of flavonols totals (mg QE/100 g dm) from grape stems from ultrasound-assisted extraction (at three acoustic power densities: 120 ± 10, 206 ± 23 and 337 ± 22 W/L) and mechanical agitation (CE, 200 rpm) at different temperatures: 10 ± 1 °C (**A**), 20 ± 2 °C (**B**) and 50 ± 4 °C (**C**) in study.

**Figure 3:**
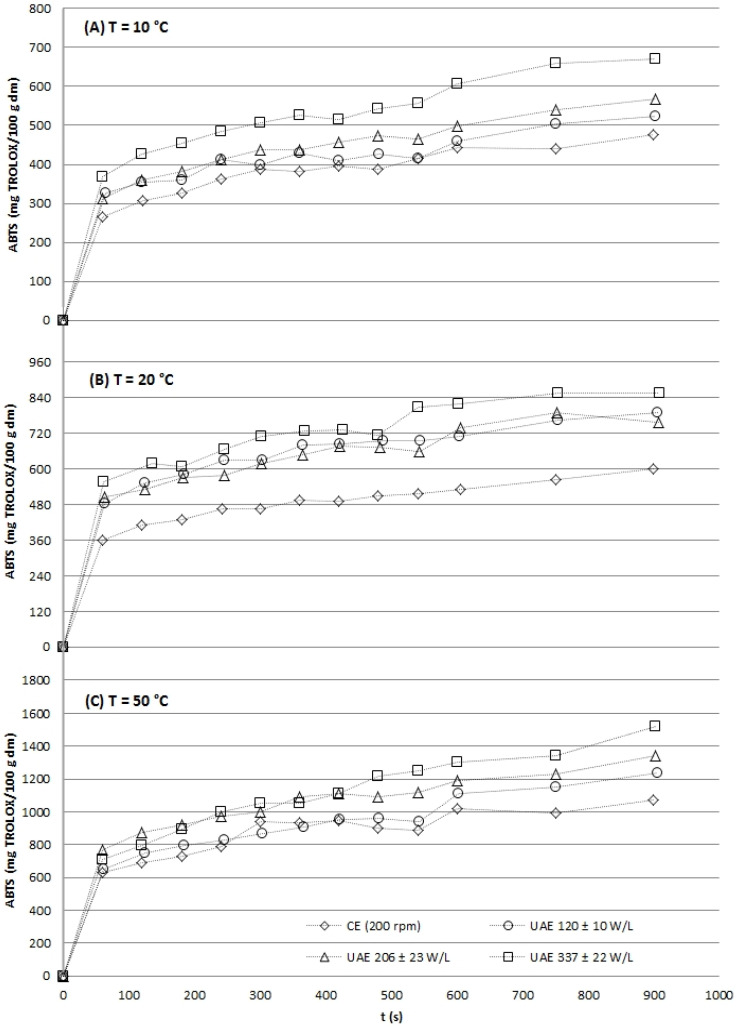
Aqueous experimental extraction curves of grape stems at different temperatures (10 ± 1 °C (**A**), 20 ± 2 °C (**B**) and 50 ± 4 °C (**C**)) under agitation (CE, 200 rpm) and acoustic conditions (at three acoustic power densities: 120 ± 10, 206 ± 23 and 337 ± 22 W/L) for antioxidant capacity according to the ABTS assay.

**Figure 4:**
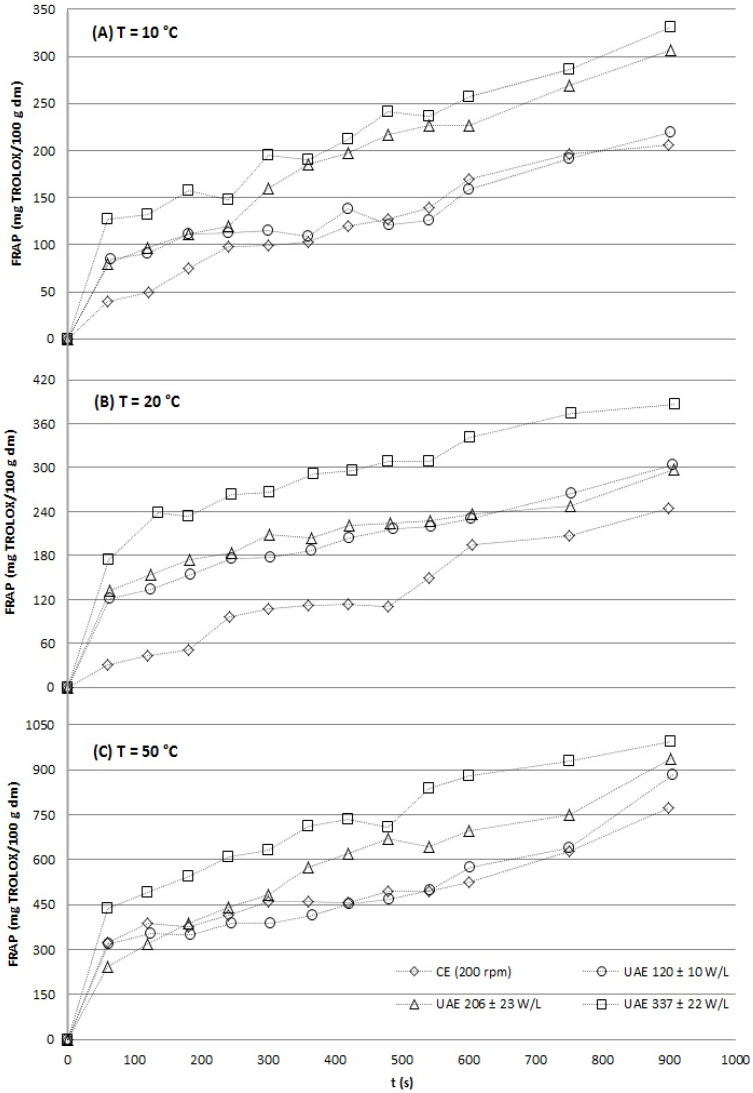
Aqueous experimental extraction curves of grape stems at different temperatures (10 ± 1 °C (**A**), 20 ± 2 °C (**B**) and 50 ± 4 °C (**C**)) under agitation (CE, 200 rpm) and acoustic conditions (at three acoustic power densities: 120 ± 10, 206 ± 23 and 337 ± 22 W/L) for antioxidant capacity according to the FRAP assay.

The kinetic profiles unequivocally demonstrated that UAE significantly enhanced both the initial extraction rate and the final yield for all target compounds: TP (Figure 1), flavonols (Figure 2), and antioxidant capacity (Figure 3 and Figure 4). At every temperature, UAE outperformed CE, achieving higher yields in a shorter timeframe. 

This enhancement is primarily attributed to the physical mechanisms of acoustic cavitation [13,27]. The implosion of cavitation bubbles generates intense shear forces, micro-jets, and shockwaves that physically disrupt cell walls, drastically increase the solid–liquid interfacial area, and promote deeper solvent penetration into the plant matrix. This accelerates mass transfer, evidenced by the characteristically steeper initial slopes of the extraction curves under sonication.

However, the efficacy of ultrasound interacts with temperature. While elevated temperature generally improves solubility and diffusion, it also increases the solvent’s vapor pressure, which can cushion bubble collapse and attenuate cavitation intensity, thereby reducing its mechanical effectiveness [27,32]. This interaction explains the observed temperature-dependent efficacy of UAE. The strong correlation between the recovery of phenolic compounds and the measured antioxidant capacity confirms that the extracted phenolics are the primary contributors to the free radical scavenging activity of the grape stem extracts [33].

#### 2.2.1. Total Phenolics

The aqueous extraction kinetics of total phenolics (TP) from grape stems (Figure 1) revealed that ultrasound-assisted extraction (UAE) significantly enhanced yields compared to conventional extraction (CE) across all tested temperatures (10, 20, and 50 °C).

The magnitude of this enhancement was temperature-dependent; it was most pronounced at 20 °C, where high-power UAE (337 W/L) yielded a 3.1-fold higher TP concentration than CE after just one minute, and less distinct at 50 °C. This attenuation at higher temperature is consistent with the known effects of increased solvent vapor pressure, which cushions cavitation bubble collapse and reduces the mechanical efficiency of ultrasound [13,27].

Furthermore, within UAE treatments, a positive correlation was observed between applied ultrasonic power density (from 120 to 337 W/L) and TP recovery, aligning with the principle that greater acoustic energy generates more cavitation events for superior cell wall disruption [34]. As expected, TP yields increased with both extraction time and temperature for all methods, with the maximum yield (1127.0 ± 12.2 mg GA/100 g dm) achieved under the most intensive conditions: UAE at 337 W/L and 50 °C for 15 min. The synergistic effect of moderate heat and acoustic energy for phenolic recovery from grape by-products has been previously reported [35,36], confirming that UAE is a highly effective strategy for valorizing grape stems, with optimal efficiency observed at 20 °C.

#### 2.2.2. Flavonol Totals

The aqueous extraction kinetics of total flavonols (quantified as quercetin equivalent) from grape stems are shown in Figure 2.

Ultrasound-assisted extraction (UAE) significantly enhanced flavonol recovery, particularly at lower temperatures. At 10 °C, UAE increased yields by approximately 1.4-fold compared to conventional extraction (CE) after 15 min, with no significant difference observed between the applied acoustic powers (120–337 W/L).

Notably, UAE achieved 50% of the maximum extractable flavonols within the first 60 s, whereas CE required longer to reach 70% recovery. At 20 °C, the effect of ultrasound was less distinct at lower power densities, but the highest power (337 W/L) produced flavonol yields comparable to those obtained by CE at 50 °C, demonstrating that UAE can achieve similar efficiency at substantially lower temperatures, thereby reducing thermal degradation risk [13,27]. At 50 °C, thermal effects dominated, though UAE at higher power densities still provided a modest but significant improvement over CE. Across all temperatures, increasing ultrasonic power density positively influenced extraction rates, which is attributed to enhanced cavitation-driven cell wall disruption and improved mass transfer [34,37].

These findings align with previous studies on plant matrices such as apple pomace and soy, where UAE improved flavonoid yields by 15–50% and substantially reduced required extraction times [38,39,40].

#### 2.2.3. Antioxidant Activity

The antioxidant capacity of grape stem extracts, determined by ABTS and FRAP assays, increased with extraction time and temperature under all conditions, though ultrasound-assisted extraction (UAE) significantly enhanced both the rate and final yield (Figure 3 and Figure 4).

At 10 °C, UAE at the highest power (337 W/L) yielded the greatest antioxidant activity after 15 min (670.9 and 331.3 mg Trolox/100 g dm by ABTS and FRAP, respectively), significantly outperforming conventional extraction (CE) and low-power UAE (120 W/L). This demonstrates that sufficient acoustic energy can effectively compensate for low thermal energy in liberating antioxidants. At 20 °C, UAE drastically reduced the time needed to match CE’s final antioxidant capacity; for instance, UAE reached in approximately 180 s what CE achieved in 900 s.

Notably, at this temperature, the highest UAE power (337 W/L) produced antioxidant levels comparable to those obtained by CE at 50 °C, reinforcing that UAE can achieve high efficiency at significantly lower, more compound-preserving temperatures [13,27]. At 50 °C, the thermal effect dominated, diminishing the relative improvement from ultrasound, though high-power UAE still reduced the required extraction time by approximately half compared to CE. While statistically significant (*p* < 0.05), the improvement at 50 °C was marginal compared to the dominant effect of thermal energy. The key practical advantage across all temperatures was the drastic reduction in extraction time.

Across all temperatures, a clear positive correlation was observed between ultrasonic power density and antioxidant recovery rate, attributable to enhanced cell wall disruption via cavitation [34]. Crucially, UAE reduced extraction times by 3- to 6-fold to achieve phenolic and antioxidant yields equivalent to CE, a well-documented efficiency gain for plant matrices like citrus peels and apple pomace [37,38].

These results confirm UAE as a highly effective, low-temperature strategy for rapidly extracting antioxidant-rich fractions from grape stems, aligning with circular bioeconomy goals by adding value to this underutilized by-product.

#### 2.2.4. Process Efficiency

To fully quantify the process intensification achieved by UAE, the operation time required for conventional extraction (CE) to obtain bioactive yields equivalent to those achieved by UAE in a fixed period of 15 min was determined and is presented in Table 2. This comparative analysis reveals the most compelling practical advantage of the ultrasonic process.

The data in Table 2 demonstrates that UAE confers a dramatic reduction in necessary extraction time. Across all temperatures and for all measured compounds, total phenolics (TP), flavonols, and antioxidant capacity (ABTS, FRAP), CE required 3 to over 6 times longer to match the yields obtained by UAE in just 15 min. For instance, to achieve the total phenolic (TP) yield obtained at 20 °C and 337 W/L by UAE in 15 min (797.49 mg GA/100 g dm), CE required over 90 min, a time reduction factor of at least six. Similarly, matching the high antioxidant capacity (ABTS: 670.88 mg Trolox/100 g dm; FRAP: 331.25 mg Trolox/100 g dm) obtained at 10 °C and 337 W/L required CE to operate for 70 and 50 min, respectively, representing 4.7- and 3.3-fold longer durations.

This efficiency gain is power-dependent. As the ultrasonic power density increased from 120 to 337 W/L, the time required for CE to match the 15 min UAE yield generally increased substantially for all bioactive metrics. This indicates that higher-power UAE extracts compounds so rapidly that CE must work exponentially longer to compensate, highlighting the intensified mechanical action of powerful acoustic cavitation.

Furthermore, Table 2 provides direct evidence that UAE enables high-efficiency extraction at low, preservation-friendly temperatures. A key finding is that UAE at 20 °C and high power (337 W/L) achieved TP, flavonol, and antioxidant yields that CE could not match even with extended processing (>90 min for TP) at the same temperature. To attain similar yields, CE would necessitate a shift to much higher temperatures (e.g., 50 °C), which carries a greater risk of thermal degradation for heat-sensitive antioxidants and polymers. This decoupling of high yield from high thermal input is a fundamental advantage of the UAE process.

The compound-specific data also reveals slightly different extraction kinetics; the time for CE to match UAE’s flavonol and FRAP antioxidant yields was often shorter than for TP or ABTS at the same condition, suggesting antioxidant compounds may be marginally more accessible or that UAE is exceptionally effective at liberating the broader phenolic matrix responsible for the bulk of the radical scavenging activity (ABTS).

To the best of the authors’ knowledge, this is one of the first studies to systematically compare conventional and acoustic extraction from winery by-products using water, while assessing different ultrasound power densities and temperatures. The acoustic assistance and increased power density were found to significantly accelerate extraction kinetics, enhancing efficiency and yield, and reducing extraction times even at 10 °C. In contrast, at 50 °C, the differences between methods were smaller, as the dominant thermal effect attenuated the relative mechanical advantage of ultrasound [13,27,32].

**Table 2:** Operation time required for conventional extraction (CE) to obtain extracts with total phenolics (TP), flavonol totals and antioxidant activity (ABTS and FRAP) similar to those obtained for extracts derived from ultrasound-assisted extraction (UAE) after 15 min.

Temperature (°C)	US Power Density (W/L)	TP_UAE at 15 min_ (mg GA/100 g dm)	* Time_CE_ to Obtain TP_UAE at 15 min_ (min)	Flavonols_UAE at 15 min_ (mg QE/100 g dm)	Time_CE_ to Obtain Flavonols_UAE at 15 min_ (min)
	120 ± 10	326.05 ± 20.61	20	12.56 ± 0.75	45
10	206 ± 23	434.02 ± 14.39	40	12.92 ± 1.68	50
	337 ± 22	486.63 ± 12.79	50	13.08 ± 1.44	50
	120 ± 10	538.31 ± 57.85	45	19.08 ± 2.03	15
20	206 ± 23	598.11 ± 29.84	60	19.42 ± 1.64	15
	337 ± 22	797.49 ± 18.27	>90	33.79 ± 3.11	30
	120 ± 10	928.03 ± 49.10	30	27.00 ± 2.28	10
30	206 ± 23	1049.84 ± 61.00	45	34.19 ± 3.35	25
	337 ± 22	1127.02 ± 12.21	55	36.29 ± 3.02	30
**Temperature** (°C)	**US power density** (W/L)	**ABTS_UAE at 15 min_** (mg TROLOX/100 g dm)	**Time**_CE_ to obtain ABTS_UAE at 15 min_ (min)	**FRAP**_UAE at 15 min_ (mg TROLOX/100 g dm)	**Time**_CE_ to obtain FRAP_UAE at 15 min_ (min)
	120 ± 10	522.33 ± 13.03	20	219.49 ± 3.91	15
10	206 ± 23	569.24 ± 36.62	20	306.44 ± 13.75	45
	337 ± 22	670.88 ± 15.64	70	331.25 ± 23.58	50
	120 ± 10	788.66 ± 34.64	20	290.58 ± 9.15	20
20	206 ± 23	790.56 ± 52.26	20	297.90 ± 25.43	25
	337 ± 22	855.73 ± 7.33	25	386.69 ± 1.31	30
	120 ± 10	1234.17 ± 32.52	20	883.88 ± 33.10	20
30	206 ± 23	1342.69 ± 20.98	25	938.31 ± 23.40	20
	337 ± 22	1520.92 ± 11.46	30	993.99 ± 27.08	25

* Time_CE_: the time required for Conventional Extraction (CE at 200 rpm) to reach the yield of the specified compound achieved by the corresponding UAE treatment in 15 min, determined by interpolation from CE kinetic curves. (“>90 min” indicates the target yield was not reached within the 90 min experiment).

The results from the extraction kinetics and the quantitative comparison in Table 2 have clear practical and economic implications. To quantitatively contextualize the efficiency of the developed UAE process, key outcomes can be compared with previous studies on grape by-products. For instance, conventional aqueous extraction of phenolics from grape pomace often requires 60–90 min at 50–70 °C to achieve yields comparable to those we report [41]. Our UAE process achieved equivalent or superior phenolic and antioxidant yields in just 15 min at 20 °C, representing a substantial reduction in both time and thermal energy input. Furthermore, while many studies focus solely on phenolic recovery [6], or on polysaccharide extraction via harsher chemical treatments [20], this work demonstrates the unique advantage of a simultaneous, one-step co-extraction in water, producing a multifunctional matrix not previously reported for grape stems. From an environmental and waste valorization perspective, this can reduce overall energy and operational costs while upgrading the value of abundant raw materials like grape stems.

## 3. Conclusions

This study establishes ultrasound-assisted aqueous extraction (UAE) as an efficient process aligned with green chemistry principles for the integrated valorization of grape stems, transforming them into optimized extract precursors for composite hydrogels with enhanced antioxidant properties.

UAE dramatically intensified the process, reducing extraction time by 3- to over 6-fold compared to conventional methods and achieving high phenolic and antioxidant yields at a mild 20 °C equivalent to those from thermal extraction at 50 °C, thereby minimizing thermal degradation risk.

While maximizing total polysaccharide yield at 10 °C, UAE’s primary effect was the facilitation of extraction and potential modification of the co-extracted polymers, predominantly pectins and hemicelluloses, which, together with soluble glucans (~49–52 mol%), form a composite polysaccharide matrix. This composite extract matrix, combining phenolics and polysaccharides, exhibits high antioxidant capacity, providing a foundational material for future development of bioactive gels.

The use of water, low temperatures, and reduced time aligns the process with green chemistry principles, establishing a sustainable foundation for valorization. The work demonstrates a sustainable, one-step route to produce value-added ingredients from wine by-products, aligning with circular bioeconomy principles for applications in food and pharmaceuticals.

## 4. Materials and Methods

### 4.1. Samples

In this study, grape stems (*Vitis vinifera* L.) provided by Ca’n Majoral winery (Mallorca, Balearic Islands, Spain) were obtained the day of grape harvest after the destemming process. Syrah grape stems were selected as a model feedstock due to their global relevance as a major wine by-product, their well-characterized cell wall architecture rich in both structural polysaccharides and phenolics [28] and the clear industrial opportunity for valorization within the wine sector.

The stem samples were dried to a final moisture of 0.70 H_2_O/g dm to ensure their stability until extraction and analysis [42]. Dried stems were stored at room temperature in darkness and protected from air moisture until further analysis. Grape stems of approximately 1 cm length were used in all experiments [43].

### 4.2. Ultrasound-Assisted Extraction Process

The experimental setup used to carry out acoustic extractions was similar to that previously described by González-Centeno et al. [35] with certain modifications. Ultrasound-assisted extractions (UAEs) were performed using three different power densities (120 ± 10, 206 ± 23, and 337 ± 22 W/L) for each temperature tested, following a factorial design to evaluate parameter interactions [44]. The US device consisted of a probe system (UP400S, Hielscher Ultrasound Technology, Teltow, Germany) of 22 mm in diameter, working at a frequency of 55 ± 5 kHz and cycles of 0.5 s [35]. Water was used as the extraction solvent at a ratio of 4.5:100 (*w*/*v*, g/mL), with a total extraction volume of 300 mL, in a vessel with a double glass layer at different controlled temperatures (10 ± 1, 20 ± 2, and 50 ± 4 °C) [27].

In this case, aliquot samples (1 mL) were taken during the first 15 min of the process. Acoustic extractions were performed in triplicate for each US power density × temperature condition tested [13].

All extracts were filtered (RC-membrane, 0.45 μm, Sartorius Stedim Biotech GmbH, Göttingen, Germany) and stored in Eppendorf tubes at 4 °C until analysis [45].

#### Acoustic Field Characterization

For the UAE, a calorimetric study was performed to calculate the acoustic power input into the system. The calorimetric study was carried out as described by González-Centeno et al. [35] in the absence of samples and without thermostating the system, a standard method for determining effective ultrasonic power [46]. The effective US power applied (*P*) was calculated according to Equation (1) from the time–temperature curve determined experimentally. All measurements were performed in triplicate.

(1) $$P\left(W\right)=m_{sol}\cdot \;c_{e_{\left(sol\right)}}\cdot \;\frac{∆T}{∆t}$$

Acoustic power density used in this study (W/L) was defined as the *P* (W) experimentally applied divided by the total extraction volume of solvent (L) [34].

This calorimetric method determines the total acoustic power input into the liquid system. It is recognized that the presence of solid plant material may attenuate the acoustic field. Nonetheless, the reported power densities provide a consistent and reproducible basis for comparing the relative intensity of different UAE treatments within this study.

### 4.3. Conventional Extraction

For comparison purposes, conventional extractions (CEs) were carried out with mechanical agitation, using a stirrer (RZR 2021, Heidolph, Schwabach, Germany) equipped with a 4-blade propeller (50 mm diameter, 200 rpm) [46]. Aliquot samples (1 mL) were taken at preset times (from 1 to 90 min) [47,48]. All extractions, either UAE or CE, were carried out in triplicate.

### 4.4. Alcohol-Insoluble Residues (AIRs) and Analysis of Carbohydrate Composition

The isolation of cell wall material from grape stems, either fresh or processed, was carried out by immersing the samples in boiling ethanol (final concentration 85% (*v*/*v*) aq.) as described by González-Centeno et al. [28], a standard procedure for obtaining alcohol-insoluble residues rich in cell wall polysaccharides [49]. This analysis provides an indirect quantification and compositional profile of the cell wall polysaccharides solubilized during extraction, inferred from the difference between the fresh and post-extraction stem AIRs.

Prior to further analysis, AIRs were ground and passed through a 0.5 mm aperture sieve [28].

To determine the carbohydrate composition of cell wall polysaccharides present in the AIRs, neutral sugars were released by acid hydrolysis following the Saeman method [50]. The released monosaccharides were converted into their alditol acetates and separated by gas–liquid chromatography at 220 °C on a 3% OV225 Chromosorb WHP 100/120 mesh column (Hewlett-Packard 5890A, Waldbronn, Germany) as described by González-Centeno et al. [28].

Uronic acids were determined by colorimetry as total uronic acids using a sample hydrolyzed for 1 h at 100 °C in 1 M H_2_SO_4_, based on the method of Blumenkrantz and Asboe-Hansen [14,42]. All results given for carbohydrate composition correspond to at least duplicate determinations.

### 4.5. Determination of Bioactive Compounds from Grape Stem Extracts

#### 4.5.1. Determination of Total Phenolics

Total phenolics (TP) of all stem extracts were spectrophotometrically measured according to the Folin–Ciocalteu method, with certain modifications adapted to analysis in 96-well microplates [34]. An automated microplate reader MultiSkan Spectrum (Thermo Fisher Scientific, Vantaa, Finland) was used for the measurement. TP results were expressed as mg of gallic acid (GA) per 100 g of stem sample (on a dry matter basis, dm) [30,31]. Each result is the mean of six determinations.

#### 4.5.2. Determination of Flavonols Content

Flavonols were determined by measuring the absorbance of the sample at 370 nm and expressed as mg of quercetin (QE) per 100 g of stem sample (dry matter basis, dm) [32]. The analysis was conducted at 25 °C using the microplate spectrophotometer mentioned above. Quercetin (0–100 µM) was used as a standard for calibration through a calibration curve [32]. Six replicates were performed for each grape stem extract.

### 4.6. Antioxidant Capacity of Grape Stem Extracts

Both ABTS and FRAP assays were performed to evaluate the antioxidant capacity of the extracts, according to the procedures previously described by González-Centeno et al. [34] and based on established protocols [35,36]. In both cases, the MultiSkan Spectrum spectrophotometer mentioned above was used. The difference in absorbance between a final reading and the reagent blank reading was correlated with Trolox standard curves (0–1.3 mM). The antioxidant capacity results were expressed as mg of Trolox per 100 g of dry matter basis (dm) [34]. Each value is the mean of six experimental determinations.

### 4.7. Statistical Analysis

All experimental results are reported as mean values with their corresponding standard deviations. Statistical significance was assessed using analysis of variance (ANOVA) with a post hoc Tukey test (*p* < 0.05) [49]. All extractions and analyses were performed in triplicate (n = 3). Triplicate independent experiments are standard for comparative screening studies of this scale and complexity, providing sufficient data to estimate variance, perform statistical tests on endpoint yields, and establish the major comparative trends between UAE and conventional methods.

## Figures and Tables

**Table 1:** Carbohydrate analysis of fresh and processed stems (CE: conventional extraction, UAE: ultrasonic-assisted extraction).

		10 °C				20 °C				50 °C			
Fresh Stems		CE	UAE_120_	UAE_206_	UAE_337_	CE	UAE_120_	UAE_206_	UAE_337_	CE	UAE_120_	UAE_206_	UAE_337_
Rha	0.9	1.0	0.9	0.9	0.8	0.9	0.7	0.9	0.8	0.7	0.6	0.5	0.4
Fuc	0.4	0.5	0.5	0.4	0.5	0.5	0.4	0.5	0.4	0.6	0.6	0.4	0.5
Ara	4.1	4.5	4.9	4.4	4.5	4.3	4.5	4.3	4.6	4.2	4.1	4.0	4.1
Xyl	15.9	15.5	15.7	15.5	15.1	15.3	15.6	15.1	15.2	15.2	14.8	14.7	14.9
Man	1.9	1.6	1.8	1.7	1.9	2.3	2.1	2.2	2.0	1.2	1.3	1.8	1.4
Gal	2.9	3.5	2.9	3.4	2.5	3.3	3.5	4.2	4.0	3.7	4.4	3.9	3.8
Glc	49.5	49.7	51.2	52.3	52.5	49.5	49.9	50.5	51.2	49.9	49.5	50.5	50.9
Glc (1M)	(4.9)	(4.5)	(5.1)	(4.4)	(4.5)	(4.6)	(4.9)	(4.4)	(4.5)	(4.4)	(4.5)	(4.7)	(4.3)
Uronic A.	24.4	23.7	22.1	21.4	22.2	23.9	23.3	22.3	21.8	24.5	24.7	24.2	24.0
Total sugars *	*18.9*	*18.7*	*19.2*	*18.7*	*17.2*	*17.9*	*17.3*	*17.0*	*16.8*	*16.7*	*15.8*	*15.4*	*14.7*

* Individual sugars are expressed in mol%, whereas total sugars are given as g/100 fresh weight.

## Data Availability

The original contributions presented in this study are included in the article. Further inquiries can be directed to the corresponding author.
